# The Evolving Role of Second- and Third-Generation Tyrosine Kinase Inhibitors in Gastrointestinal Malignancies: Advances in Targeted Therapy with Sunitinib, Regorafenib, and Avapritinib

**DOI:** 10.3390/jcm15010317

**Published:** 2026-01-01

**Authors:** Piotr Kawczak, Tomasz Bączek

**Affiliations:** 1 Department of Pharmaceutical Chemistry, Faculty of Pharmacy, Medical University of Gdańsk, 80-416 Gdańsk, Poland; tomasz.baczek@gumed.edu.pl; 2 Department of Nursing and Medical Rescue, Institute of Health Sciences, Pomeranian University in Słupsk, 76-200 Słupsk, Poland

**Keywords:** tyrosine kinase inhibitors, sunitinib, regorafenib, avapritinib, gastrointestinal stromal tumor, targeted therapy, gastrointestinal malignancies, precision oncology, molecular-guided therapy

## Abstract

Gastrointestinal stromal tumors (GISTs) are the most common mesenchymal tumors of the gastrointestinal tract. While imatinib revolutionized first-line therapy, resistance and specific mutation profiles necessitate subsequent generations of tyrosine kinase inhibitors (TKIs). Sunitinib, regorafenib, and avapritinib represent second-line, third-line, and mutation-specific therapies, respectively, offering improved precision and disease control. This review summarizes clinical trial evidence, real-world data, and translational studies evaluating the efficacy, safety, and mechanistic basis of second- and third-generation TKIs in GIST. Emphasis is placed on therapeutic sequencing, resistance mechanisms, and molecularly guided treatment selection. Sunitinib, a multitargeted TKI inhibiting KIT, PDGFR, and VEGFR, provides effective disease control in imatinib-resistant or intolerant patients. Regorafenib, a broad-spectrum multikinase inhibitor, improves progression-free survival in refractory GIST and targets additional angiogenic and oncogenic pathways. Avapritinib, a next-generation TKI, selectively inhibits PDGFRA D842V and KIT exon 17 mutations, addressing a previously untreatable, mutation-driven subgroup. Integration of these agents into treatment algorithms exemplifies a shift toward personalized therapy, with outcomes guided by mutation profiling and biomarker-driven decisions. Second- and third-generation TKIs have transformed the management of advanced GIST, extending survival and offering mutation-specific precision therapy. Ongoing research into resistance mechanisms, combination strategies, and novel inhibitors promises further optimization of patient-centered care.

## 1. Introduction

Gastrointestinal stromal tumors (GISTs) are the most common mesenchymal neoplasms of the gastrointestinal tract and originate from the interstitial cells of Cajal, with most driven by activating mutations in KIT or PDGFRA [1,2,3,4]. As the prototypical and most extensively characterized gastrointestinal mesenchymal tumor, GIST occupies a distinct position among gastrointestinal malignancies—most of which are epithelial cancers such as gastric and colorectal adenocarcinomas or neuroendocrine tumors, lymphomas, and other soft-tissue sarcomas [5,6,7].

The majority of GISTs are driven by sporadic somatic KIT or PDGFRA mutations rather than inflammation-associated carcinogenesis; rare hereditary and syndromic forms of GIST (e.g., familial KIT/PDGFRA mutations, Carney–Stratakis syndrome, and neurofibromatosis type 1–associated GIST) do exist [8,9,10]. The identification of KIT and PDGFRA mutations fundamentally reshaped the biological classification and clinical management of GIST, distinguishing it from other sarcomas and enabling molecularly targeted therapeutic development [11,12,13]. This foundation positioned GIST as an early model of precision oncology within solid tumors and underscored the importance of genotype-directed therapy in gastrointestinal cancer care.

Tyrosine kinase inhibitors (TKIs) have transformed the treatment of GIST and other gastrointestinal malignancies, including metastatic colorectal cancer (mCRC), through selective targeting of oncogenic kinases. These agents are commonly categorized into first-, second-, and third-generation inhibitors based on progressive refinements in potency, selectivity, and activity against resistance mutations [14,15,16]. Multitargeted TKIs such as sunitinib, regorafenib, and sorafenib—originally developed and validated in renal cell carcinoma, hepatocellular carcinoma, and mCRC—exert antitumor effects primarily via inhibition of VEGFR, PDGFR, RAF, and KIT-related signaling pathways. Because these pathways are central to GIST pathogenesis and the emergence of imatinib resistance, insights from angiogenesis-driven malignancies have informed both the mechanistic rationale and clinical sequencing of TKIs in GIST [7,15,17]

First-generation TKIs such as imatinib, which inhibit KIT, PDGFR, and BCR-ABL, revolutionized first-line therapy for GIST and chronic myeloid leukemia (CML) [18,19,20]. Imatinib achieved unprecedented disease control in advanced GIST, which had historically been refractory to cytotoxic chemotherapy and radiotherapy [21,22,23]. However, primary resistance—particularly in tumors harboring the PDGFRA D842V mutation—and the near-universal emergence of secondary resistance mutations during treatment ultimately limited long-term efficacy [24,25,26]. These resistance dynamics parallel clonal evolution observed across advanced gastrointestinal malignancies, where selective pressure drives molecular adaptation and treatment failure.

Second-generation TKIs were developed to counter resistance and broaden inhibitory profiles. Multitargeted agents such as sunitinib [27], regorafenib [28], sorafenib [29], pazopanib [30], cabozantinib [31], and axitinib [32] inhibit a range of kinases, including VEGFR, PDGFR, KIT, and RET, producing both antitumor and antiangiogenic effects. These agents have demonstrated substantial benefit in imatinib-resistant GIST and in malignancies such as refractory mCRC, renal cell carcinoma (RCC), and hepatocellular carcinoma (HCC) [27,28,29,30,31,32]. Sunitinib became standard second-line therapy after improving outcomes in patients progressing on imatinib [33,34,35], with differential efficacy across molecular subsets—including enhanced activity in tumors with KIT exon 9 mutations and specific secondary mutations [36,37]—highlighting the heterogeneity of GIST evolution and reinforcing the need for molecular profiling.

Regorafenib, a broad-spectrum inhibitor of angiogenic and oncogenic kinases including TIE2, RAF, RET, and FGFR, further extended therapeutic options in the third-line therapy setting. The phase III GRID trial established its efficacy in delaying disease progression following imatinib and sunitinib failure [38,39,40], and subsequent real-world studies confirmed its clinical utility and manageable toxicity across heterogeneous patient populations [41,42]. In this context, the term “diverse patient groups” encompasses heterogeneity in age, KIT/PDGFRA mutational profiles, extent of prior TKI exposure, and geographic background, as reflected in both pivotal clinical trials and post-approval cohorts [37,38,43]. Within the biological landscape of late-line GIST—characterized by the accumulation of multiple co-existing secondary resistance mutations—regorafenib’s activity across angiogenic and oncogenic signaling pathways has demonstrated consistent benefit despite increasing molecular complexity.

The advent of third-generation TKIs marked a shift toward mutation-specific precision therapy. Molecular analyses of imatinib-resistant GIST have demonstrated that secondary KIT mutations arise predominantly within the ATP-binding pocket (exons 13 and 14) or the activation loop (exons 17 and 18), with these distinct alterations differentially affecting sensitivity to subsequent TKIs [44,45].

Against this backdrop, avapritinib emerged as a highly potent type I inhibitor engineered to selectively target activation-loop mutations, including PDGFRA D842V and KIT exon 17 variants, providing a therapeutic breakthrough for molecular subsets previously resistant to all available TKIs [46,47,48,49]. Results from the NAVIGATOR and VOYAGER trials demonstrated exceptional efficacy in PDGFRA D842V-mutant GIST [50,51], leading to global regulatory approval.

**Figure 1:**
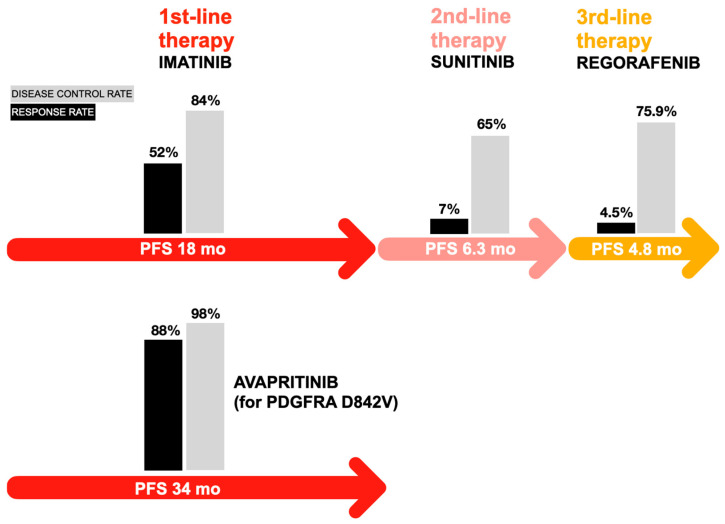
Treatment strategies using FDA-approved tyrosine kinase inhibitors: imatinib, sunitinib, regorafenib, and avapritinib for advanced gastrointestinal stromal tumors according to [60], where mo—months and PFS—Progression-Free Survival.

As GISTs progress through successive lines of therapy, increasing molecular heterogeneity driven by the accumulation of secondary KIT mutations becomes a defining feature of late-line disease [52,53]. While earlier multitargeted TKIs such as sunitinib and regorafenib provide partial coverage of resistance mutations, their efficacy is often limited by differential sensitivity across ATP-binding pocket (exons 13/14) and activation loop (exons 17/18) alterations [52,53]. This therapeutic challenge prompted the development of next-generation inhibitors designed to more comprehensively suppress a broad spectrum of KIT and PDGFRA mutants. Ripretinib, a switch-control kinase inhibitor that stabilizes KIT and PDGFRA in an inactive conformation, represents a distinct mechanistic advance and has expanded treatment options for heavily pretreated patients [54,55,56]. The pivotal phase III INVICTUS trial demonstrated that ripretinib significantly improved progression-free survival compared with placebo, establishing its role as a standard late-line therapy in advanced GIST [55]. Collectively, these advances underscore the increasing importance of comprehensive molecular diagnostics in guiding mutation-tailored therapy, informing optimal treatment sequencing, and adapting therapeutic strategies as tumor biology evolves under selective pressure [57,58,59]. Table 1 summarizes first-, second-, and third-generation TKIs utilized in gastrointestinal malignancies and associated cancers, while Figure 1 depicts treatment strategies for advanced gastrointestinal stromal tumors employing FDA-approved tyrosine kinase inhibitors, including imatinib, sunitinib, regorafenib, and avapritinib.

Ongoing research into resistance mechanisms, kinase structural dynamics, and microenvironmental influences continues to inform next-generation inhibitor development and combination strategies [61,62].

The development of second- and third-generation TKIs—including sunitinib, regorafenib, and avapritinib—has markedly advanced the management of advanced GIST, allowing more precise, mutation-driven therapy and extending patient survival compared with earlier treatment eras. As precision oncology continues to evolve, the integration of molecularly targeted agents with emerging therapeutic modalities promises to further optimize care for patients with GIST.

First-generation TKI therapy (imatinib) has been extensively reviewed in the literature and is well established as first-line treatment. In contrast, therapeutic resistance to imatinib and the molecular heterogeneity of GIST have driven the development of second- and third-generation TKIs, which remain an area of rapid progress and clinical interest. Therefore, our review intentionally focuses on these later-generation agents to highlight advances in overcoming resistance mechanisms and improving precision treatment.

This review synthesizes current clinical, translational, and molecular evidence supporting precision TKI use in GIST, integrating data from pivotal trials, real-world studies, and mechanistic analyses. In addition, it highlights emerging challenges, including the management of polyclonal resistance, heterogeneity across KIT and PDGFRA mutations, and the need for dynamic monitoring of tumor evolution. By examining both established and next-generation therapies, this review emphasizes opportunities to optimize genotype-driven treatment strategies and to incorporate molecular insights into routine clinical practice, ultimately aiming to improve patient outcomes in advanced GIST.

This synthesis was informed by a narrative literature review of PubMed and Scopus using the search terms “sunitinib,” “regorafenib,” and “avapritinib” combined with “ targeted therapy” and “gastrointestinal stromal tumors.” Peer-reviewed publications from 2005 to 2025 were selected for relevance, methodological rigor, and contributions to understanding therapeutic efficacy, mechanisms of action, and resistance patterns. Both preclinical and clinical studies were included when mechanistic insights informed therapeutic strategy, enabling a comprehensive evaluation of the roles, benefits, and limitations of sunitinib, regorafenib, and avapritinib in contemporary gastrointestinal oncology.

## 2. Sunitinib

**Figure 2:**
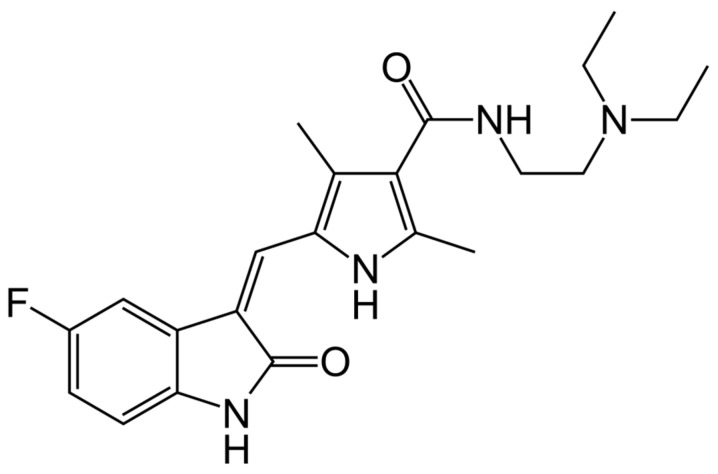
Structural formula of sunitinib.

Sunitinib is an orally bioavailable, small-molecule, multi-targeted tyrosine kinase inhibitor (TKI) designed to block key signaling pathways that regulate tumor cell proliferation, angiogenesis, and metastatic progression [14,63]. It is classified as a second-generation multitarget TKI with activity against a broad panel of receptor tyrosine kinases, including vascular endothelial growth factor receptors (VEGFR-1, VEGFR-2, VEGFR-3), platelet-derived growth factor receptors (PDGFR-α/β), and proto-oncogenic kinases such as KIT, RET, CSF-1R, and FLT3 [64,65,66]. This inhibitory spectrum enables sunitinib to simultaneously modulate angiogenic, stromal, and oncogenic signaling within the tumor microenvironment, disrupting critical processes required for tumor maintenance and dissemination [67,68,69]. Figure 2 shows structural formula of sunitinib.

**Figure 3:**
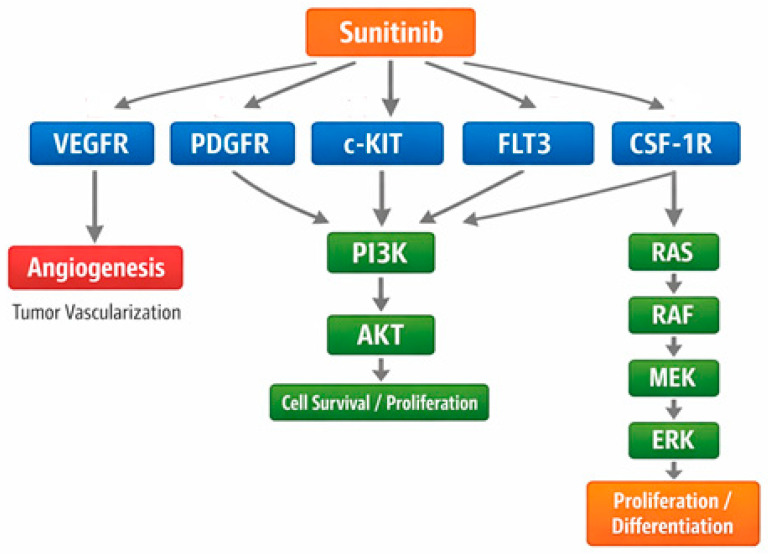
Sunitinib targets and downstream signaling pathways, where VEGFR—Vascular Endothelial Growth Factor Receptor; PDGFR—Platelet-Derived Growth Factor Receptor; c-KIT—Stem Cell Factor Receptor/KIT Proto-Oncogene; FLT3—FMS-like Tyrosine Kinase 3; CSF-1R—Colony-Stimulating Factor 1 Receptor; PI3K—Phosphoinositide 3-Kinase; AKT—Protein Kinase B; RAS—Rat Sarcoma Protein; RAF—Rapidly Accelerated Fibrosarcoma Kinase; MEK—Mitogen-Activated Protein Kinase Kinase; ERK—Extracellular Signal-Regulated Kinase.

Mechanistically, sunitinib functions as a competitive ATP-binding site inhibitor, preventing phosphorylation of downstream signaling molecules that govern cell survival (e.g., PI3K–AKT), proliferation (e.g., MAPK), endothelial cell migration, and vascular permeability [70]. By inhibiting VEGFR and PDGFR activity, sunitinib suppresses endothelial cell proliferation, reduces pericyte coverage of nascent vessels, and leads to regression and normalization of tumor vasculature [71]. Inhibition of KIT and FLT3 further impacts hematopoietic and stromal signaling, explaining both the antitumor activity in GIST and the hematologic toxicities observed clinically. This multitarget action distinguishes sunitinib from earlier angiogenesis inhibitors and contributes to a robust antitumor profile across genetically diverse malignancies. Figure 3 illustrates the molecular targets of sunitinib and the downstream signaling pathways it modulates.

Sunitinib’s initial clinical development in the early 2000s focused on metastatic renal cell carcinoma (mRCC) and gastrointestinal stromal tumors (GIST), two tumor types highly dependent on VEGF-, PDGF-, and KIT-mediated pathways [72]. In phase II and III trials in mRCC, sunitinib yielded substantial objective response rates and significantly improved progression-free survival compared with interferon-α, the prior standard of care [27]. These studies were the first to demonstrate that multitargeted angiogenesis inhibition could provide superior outcomes to immunotherapy alone in renal cancer. In GIST populations refractory to imatinib, sunitinib offered clinically meaningful disease stabilization and prolonged time to progression, highlighting its ability to overcome resistance mediated by secondary KIT mutations [33]. These pivotal data supported the drug’s initial regulatory approval in 2006 [73].

Subsequent research expanded sunitinib’s clinical utility to pancreatic neuroendocrine tumors (pNETs), where VEGF-driven angiogenesis is prominent. In a randomized trial, sunitinib significantly prolonged progression-free survival and improved disease control rates, providing a new targeted option for this previously underserved population [74]. In other malignancies—including differentiated thyroid cancer, soft-tissue sarcoma, and pediatric solid tumors—sunitinib demonstrated varying degrees of antitumor activity [75]. Nonetheless, differences in tumor biology, resistance patterns, and toxicity profiles have limited its widespread adoption beyond its primary indications [76]. Combination regimens of sunitinib with immune checkpoint inhibitors or other TKIs have shown biological synergy in preclinical studies but have often been restricted clinically due to overlapping hepatotoxicity, fatigue, and immune-mediated adverse events [77,78]. Comparative trials with next-generation agents suggest that while sunitinib retains strong efficacy, some newer TKIs may offer enhanced target specificity or reduced toxicity burdens [79].

The drug’s adverse-effect profile reflects its broad inhibitory activity. Class-related toxicities include fatigue, hypertension, mucosal inflammation, diarrhea, and dermatologic reactions, many of which derive from VEGFR/PDGFR blockade in normal tissues [80]. Chronic inhibition of thyroidal VEGF signaling is thought to contribute to the high incidence of hypothyroidism in sunitinib-treated patients, which may require thyroid hormone replacement [81]. More severe toxicities, although less frequent, include cardiotoxic events such as decreases in left ventricular ejection fraction—likely related to mitochondrial dysfunction and off-target kinase inhibition—along with hepatotoxicity and thromboembolic complications [82]. VEGF inhibition also impairs wound healing, vascular integrity, and tissue regeneration, creating risks of bleeding or delayed recovery following surgery [83]. These risks have prompted the incorporation of rigorous monitoring guidelines for blood pressure, thyroid function, cardiac status, and hepatic enzymes throughout treatment [84]. Table 2 presents treatment-emergent adverse events (TEAE) and management strategies for sunitinib.

Clinically meaningful associations have been observed between sunitinib toxicity markers and therapeutic outcomes. Treatment-emergent hypertension has been correlated with improved progression-free and overall survival, supporting its potential role as a mechanism-based biomarker of VEGF pathway inhibition [91]. Similarly, the development of hypothyroidism has been proposed as a surrogate marker for adequate systemic kinase inhibition, although prospective evidence remains limited [92]. Efforts to identify pharmacogenomic predictors of response or toxicity—such as polymorphisms in CYP3A5, ABCB1, and VEGF pathway genes—have yielded inconsistent results, suggesting multifactorial determinants of drug metabolism and resistance [93].

Beyond its approved uses, sunitinib occupies an important role in the evolution of multitargeted oncology therapeutics. It is widely employed in preclinical studies examining mechanisms of angiogenesis, tumor–stromal interactions, and acquired resistance pathways [94]. As a well-characterized benchmark TKI, sunitinib remains a reference compound in kinase-selectivity profiling, structural biology studies, and drug-development pipelines exploring next-generation VEGFR/PDGFR inhibitors [95]. Real-world longitudinal studies continue to refine their clinical positioning, providing insights into optimal dosing schedules, strategies for managing chronic toxicity, and biomarkers for patient selection [96]. Thus, sunitinib has not only shaped therapeutic paradigms within renal and gastrointestinal oncology but also contributed substantially to the broader scientific understanding of targeted multi-kinase inhibition. Table 3 illustrates major pivotal clinical trials of sunitinib.

## 3. Regorafenib

**Figure 4:**
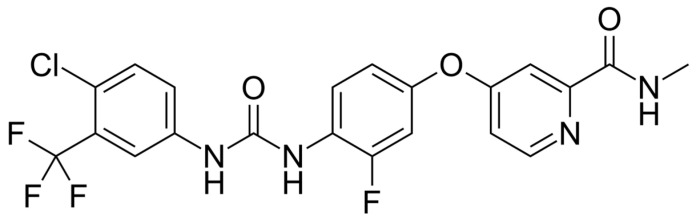
Structural formula of regorafenib.

Regorafenib is an orally administered, small-molecule multikinase inhibitor belonging to the class of second-generation angiogenesis and oncogenic signaling inhibitors [15,98]. Structurally derived from sorafenib by the addition of a fluorine atom, regorafenib was rationally designed to provide broader target selectivity and stronger potency across multiple kinase families [99,100]. Its inhibitory spectrum encompasses vascular endothelial growth factor receptors (VEGFR-1, VEGFR-2, VEGFR-3), platelet-derived growth factor receptors (PDGFR-α/β), fibroblast growth factor receptor (FGFR), KIT, RET, TIE2, DDR2, and RAF kinases [101,102]. This multilevel blockade affects both the tumor microenvironment and the cancer cells themselves, including angiogenic, stromal, and oncogenic pathways essential for tumor survival and proliferation [103,104,105]. Figure 4 depicts structural formula of regorafenib.

Mechanistically, regorafenib functions as a competitive ATP-binding inhibitor that simultaneously disrupts angiogenesis, oncogenic signaling, and metastatic processes [106]. Inhibition of VEGFR and TIE2 reduces endothelial cell proliferation, migration, and vessel maturation, thereby suppressing tumor angiogenesis and inducing vascular normalization [107]. Through PDGFR and FGFR blockade, regorafenib interferes with pericyte recruitment and fibroblast activation, weakening tumor stroma and nutrient supply [108]. Additionally, RAF/MEK/ERK pathway inhibition contributes to antiproliferative effects in tumors with MAPK dependence, although this pathway is not the primary driver of its clinical efficacy [28]. Inhibition of RET and KIT supports its therapeutic role in thyroid cancers and gastrointestinal stromal tumors (GIST), particularly in the context of resistance to earlier-generation TKIs [109]. This systems-level inhibition distinguishes regorafenib from earlier anti-angiogenic agents by expanding its activity across multiple malignant phenotypes.

Although regorafenib was initially developed for metastatic colorectal cancer (mCRC), accumulating evidence has firmly established its role in GIST, particularly in the post–imatinib and sunitinib setting. Early clinical development focused on mCRC, where resistance to chemotherapy and anti-VEGF therapies represented a major unmet need [110,111,112]. The pivotal CORRECT phase III trial demonstrated a statistically significant overall survival benefit in heavily pretreated mCRC patients, establishing regorafenib as an effective option in refractory disease [113]. This study highlighted the capacity of broad multikinase inhibition to overcome resistance mechanisms that limit the efficacy of VEGF-specific monoclonal antibodies and conventional chemotherapies [114].

Building on earlier clinical development, regorafenib was approved for GIST following failure of imatinib and sunitinib on the basis of the pivotal phase III GRID trial. In this study, regorafenib significantly improved progression-free survival compared with placebo (median PFS 4.8 vs. 0.9 months; hazard ratio 0.27), leading to its establishment as standard third-line therapy for advanced GIST [38,115]. Even though objective response rates were low, a substantial proportion of patients achieved durable disease control, consistent with the predominantly cytostatic activity of later-line tyrosine kinase inhibitors in GIST. Subsequent analyses of the GRID dataset demonstrated clinical benefit across a broad spectrum of KIT and PDGFRA mutational subtypes, including secondary mutations associated with resistance to earlier-line therapies, reinforcing regorafenib’s role as a broadly active agent in the post–imatinib and post–sunitinib setting [34,116,117]. Collectively, these findings provided definitive evidence supporting regorafenib monotherapy and solidified its position as a key component of sequential KIT-targeted therapy, as reflected in international treatment guidelines from ESMO and NCCN [118,119].

With the introduction of newer agents such as ripretinib and avapritinib, treatment sequencing beyond second line has continued to evolve. Nevertheless, regorafenib remains an integral element of contemporary GIST management owing to its proven efficacy, well-characterized safety profile, and ability to achieve durable disease stabilization in a subset of patients. Ongoing clinical experience and retrospective analyses continue to refine optimal sequencing strategies, particularly in relation to mutational status and prior treatment exposure. Beyond GIST, regorafenib’s clinical utility was further expanded in hepatocellular carcinoma (HCC), where the phase III RESORCE trial confirmed its benefit as second-line therapy after sorafenib, representing the first agent to demonstrate a survival advantage in patients progressing on prior VEGFR-targeted treatment [120].

Outside of the pivotal trials, regorafenib has been investigated across a range of tumor types, generating an expanding body of evidence with particular relevance to GIST resistance mechanisms and therapeutic sequencing. In HCC, real-world studies have corroborated survival benefits observed in randomized trials and emphasized the importance of patient selection, particularly with respect to liver function and performance status [121]. In mCRC, combination strategies incorporating regorafenib with immune checkpoint inhibitors—most notably PD-1 inhibitors—have shown encouraging activity, especially in microsatellite-stable tumors that are typically refractory to immunotherapy alone [122]. In GIST, regorafenib has demonstrated sustained benefits for patients harboring secondary KIT mutations associated with imatinib or sunitinib resistance, with continued research focusing on optimizing sequencing strategies among KIT-targeted TKIs [123]. Exploratory trials in glioblastoma, ovarian cancer, and sarcomas have shown variable results, often limited by toxicity profiles or by intrinsic tumor biology [124]. Nevertheless, regorafenib remains a benchmark multikinase inhibitor in translational oncology research.

The adverse-effect profile of regorafenib reflects its broad kinase selectivity. The most frequently reported toxicities include hand–foot skin reaction (HFSR), hypertension, fatigue, diarrhea, anorexia, and hepatotoxicity [125]. HFSR is particularly characteristic of regorafenib and occurs more frequently than with many other TKIs, likely due to off-target inhibition of kinases involved in keratinocyte function and microvascular repair [126]. Hypertension reflects on-target VEGFR blockade, while diarrhea and mucosal toxicities result from gastrointestinal epithelial injury and stromal VEGF signaling inhibition [127]. Hepatotoxicity—including elevations in transaminases and bilirubin—remains clinically significant and necessitates close monitoring, especially during the first two treatment cycles [128]. Less common but serious adverse events, such as hemorrhage, gastrointestinal perforation, and myocardial ischemia, are clinically relevant, particularly in patients with underlying vascular disease [129]. Table 4 summarizes treatment-emergent adverse events and management strategies for regorafenib.

Clinically meaningful associations between regorafenib-related toxicities and therapeutic outcomes have been reported. Early onset of HFSR has been correlated with improved survival in both mCRC and HCC, suggesting a potential exposure–response relationship indicative of adequate systemic drug levels [136]. Hypertension may similarly function as a mechanism-based biomarker of effective angiogenesis inhibition, consistent with observations across other VEGFR-targeted TKIs [137]. Pharmacogenomic investigations examining UGT1A9, ABCB1, and CYP3A4 variants have explored interindividual variability in regorafenib metabolism and toxicity, though results remain inconsistent and have not yet translated into routine clinical practice [138]. Dose-optimization studies have demonstrated that alternative dosing strategies—such as reduced starting doses with subsequent escalation—can improve tolerability without compromising efficacy, informing evolving real-world treatment approaches [139].

Complementing its approved clinical indications, regorafenib is widely employed in preclinical models to study angiogenesis, tumor–stromal interactions, and resistance to targeted therapies [140]. Its function as a reference multikinase inhibitor has made it invaluable for kinase profiling, structural analyses, and comparative drug-development research [95]. Additionally, regorafenib is increasingly evaluated in combination with immune checkpoint inhibitors, anti-FGFR agents, MAPK inhibitors, and cytotoxic chemotherapies to explore synergistic activity and overcome resistance mechanisms across diverse malignancies [141]. By integrating angiogenesis inhibition with blockade of oncogenic signaling pathways, regorafenib continues to serve as a cornerstone agent in the evolving landscape of multikinase therapeutics. Table 5 summarizes the major pivotal clinical trials of regorafenib.

## 4. Avapritinib

**Figure 5:**
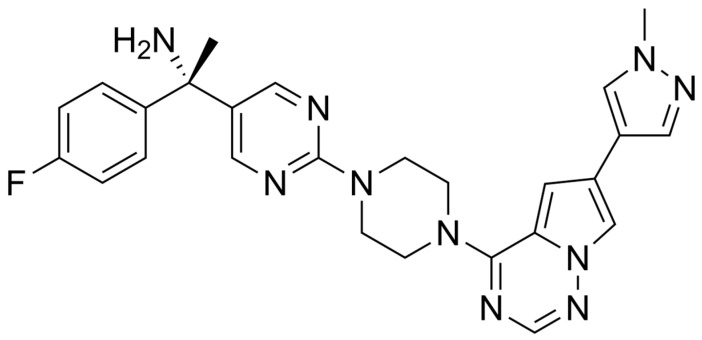
Structural formula of avapartinib.

Avapritinib is an orally administered, selective small-molecule inhibitor of receptor tyrosine kinases, specifically engineered to target oncogenic mutations in KIT and platelet-derived growth factor receptor alpha (PDGFRA) [146,147,148]. Unlike broader-spectrum tyrosine kinase inhibitors such as sunitinib or regorafenib, avapritinib was designed to exhibit high selectivity and potency against mutant forms of PDGFRA, particularly the D842V mutation, which is associated with primary resistance to conventional TKIs [47,149,150]. Its specificity minimizes off-target inhibition while maximizing activity against resistant gastrointestinal stromal tumor (GIST) clones, distinguishing it within the therapeutic landscape of TKI therapy [151,152]. Figure 5 illustartes structural formula of avapartinib.

Mechanistically, avapritinib functions as a type I ATP-competitive inhibitor that preferentially binds to the active conformation of KIT and PDGFRA mutants, blocking phosphorylation and downstream MAPK, PI3K–AKT, and STAT signaling pathways [153]. By directly inhibiting mutant-driven oncogenic signaling, avapritinib suppresses cell proliferation, promotes apoptosis, and reduces tumor vascular support indirectly, given that PDGFRA-mutant tumors often induce pro-angiogenic microenvironments [154]. The high affinity for activation-loop mutations enables the drug to overcome resistance that commonly develops with first- and second-generation TKIs in GIST [155].

Avapritinib’s clinical development initially targeted patients with unresectable or metastatic GIST harboring PDGFRA exon 18 mutations—particularly D842V—a population historically refractory to imatinib, sunitinib, and regorafenib [47,156,157]. The pivotal phase I NAVIGATOR trial assessed dose escalation, safety, and efficacy across multiple lines of prior therapy with a strong emphasis on molecularly defined subgroups, establishing 300 mg once daily as the recommended phase II dose [158]. In patients with PDGFRA exon 18 mutations, including D842V, avapritinib demonstrated remarkable clinical activity characterized by high objective response rates, deep and durable responses, and prolonged progression-free survival regardless of prior treatment exposure [43,158]. These outcomes led to accelerated regulatory approval of avapritinib for PDGFRA exon 18–mutant GIST and underscored the importance of molecular profiling for patient selection [159]. Phase II expansion cohorts further confirmed robust efficacy across PDGFRA-mutant populations with generally manageable toxicity profiles [160]. NAVIGATOR also showed activity in heavily pretreated KIT-mutant GIST, particularly in tumors with activation loop mutations, although dose-dependent neurocognitive adverse events were observed, informing subsequent dose-modification strategies and clinical management recommendations [158].

The phase III VOYAGER trial directly compared avapritinib with regorafenib in patients with advanced GIST who had received at least two prior tyrosine kinase inhibitors. Although avapritinib did not significantly improve progression-free survival compared with regorafenib in the overall study population, exploratory subgroup analyses suggested differential efficacy according to mutational status [161]. Importantly, VOYAGER confirmed regorafenib as the preferred third-line therapy for unselected GIST, while further refining the role of avapritinib as a mutation-driven therapy rather than a broadly applicable later-line agent.

In the aggregate, findings from the NAVIGATOR and VOYAGER trials highlight the critical importance of molecular stratification in GIST and clarify the optimal clinical use of avapritinib. Current international guidelines recommend avapritinib primarily for patients with PDGFRA exon 18–mutant GIST, while alternative tyrosine kinase inhibitors remain preferred in KIT-mutant disease in later-line settings [57,119].

Subsequent studies explored avapritinib in other clinical contexts. For example, patients with GIST harboring secondary KIT mutations resistant to earlier TKIs exhibited variable responses, indicating that avapritinib’s activity is strongest against specific PDGFRA activation-loop mutations [162]. Additionally, avapritinib is being investigated in combination with other targeted therapies or in early lines of therapy to determine whether synergistic antitumor effects can broaden its clinical utility [163]. Preclinical models suggest potential activity in rare KIT-driven malignancies beyond GIST, including systemic mastocytosis and certain melanomas, although clinical translation remains limited [164].

The adverse-effect profile of avapritinib is generally favorable compared with multikinase TKIs, reflecting its high selectivity, but it is not without significant toxicities [165]. Common adverse events include nausea, fatigue, anemia, edema, and cognitive effects such as memory impairment and confusion, collectively referred to as “cognitive adverse events” [166]. Intracranial hemorrhage and severe cutaneous reactions have been reported in rare cases, requiring careful monitoring [167]. Dose modifications or temporary interruptions are frequently employed to manage these toxicities without compromising efficacy [168]. Table 6 presents TEAE and management strategies for avapartinib.

Several clinically relevant associations have been observed. Rapid onset of response in PDGFRA D842V-mutant GIST is highly predictive of durable disease control, highlighting the mutation as both a therapeutic target and a predictive biomarker [172]. Cognitive adverse events may correlate with drug exposure and can be partially mitigated with dose adjustment [173]. Pharmacokinetic studies indicate that food intake increases systemic exposure modestly, which can inform administration recommendations [174].

Avapritinib has additional mentions in preclinical and translational research. Its high selectivity makes it a useful tool for dissecting the biology of PDGFRA-driven tumors and understanding mechanisms of resistance [175]. The drug is also under investigation in combination with KIT inhibitors, MEK inhibitors, or immune checkpoint blockade in early-phase trials to expand its utility beyond PDGFRA D842V-mutant GIST [176]. As a precision TKI, avapritinib represents a paradigm shift in targeted oncology, emphasizing the importance of mutation-specific drug design and personalized therapy approaches. Table 7 shows major pivotal clinical trials of avapartinib.

## 5. Emerging Trends and Opportunities

**Figure 6:**
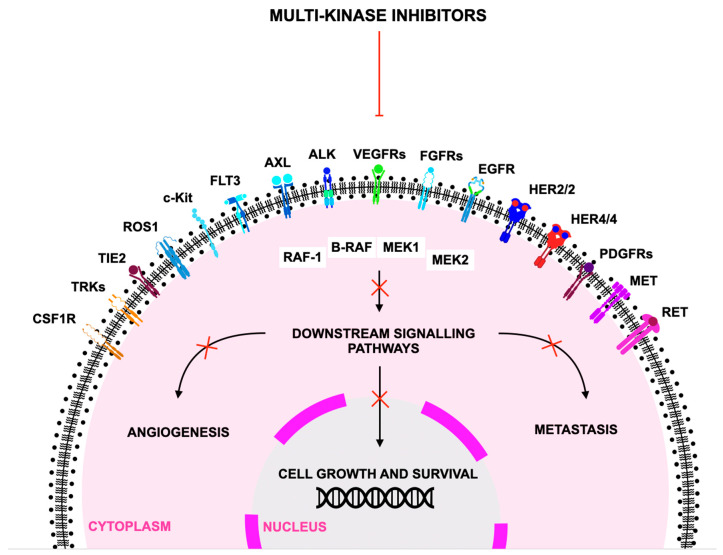
Schematic presentation of the action mechanism of multi-kinase inhibitors acting on several related kinase pathways according to [180], where ALK—Anaplastic Lymphoma Kinase; AXL—AXL receptor tyrosine kinase; B-Raf—serine/threonine-protein kinase B-Raf; c-Kit—mast/stem cell growth factor receptor; CSF1R—colony-stimulating factor 1 receptor; EGFR—epidermal growth factor receptor; FGFRs—fibroblast growth factor receptors; FLT3—FMS-like tyrosine kinase 3; HER2/2—human epidermal growth factor receptor 2; HER4/4—human epidermal growth factor receptor 4; MEK1—mitogen-activated protein kinase kinase 1; MEK2—mitogen-activated protein kinase kinase 2; MET—mesenchymal—epithelial transition factor; PDGFRs—platelet-derived growth factor receptors; Raf-1—RAF serine/threonine-protein kinase; RET—receptor tyrosine kinase rearranged during transfection; ROS1—proto-oncogene tyrosine-protein kinase ROS; TIE2—tunica interna endothelial cell kinase 2; TRKs—tropomyosin receptor tyrosine kinases; VEGFRs—vascular endothelial growth factor receptors.

The development of second- and third-generation tyrosine kinase inhibitors (TKIs) such as sunitinib, regorafenib, and avapritinib has markedly transformed the management of gastrointestinal malignancies, particularly in gastrointestinal stromal tumors (GIST) and colorectal cancer (CRC) [178]. Despite substantial advances, emerging trends suggest that the full potential of these agents is still being realized, offering opportunities for improved clinical outcomes and precision therapy [47,179]. Figure 6 illustrates how multi-kinase inhibitors function by targeting a spectrum of related kinases.

One prominent trend is the rational design of mutation-specific TKIs, exemplified by avapritinib. By selectively targeting PDGFRA D842V and other activation-loop mutations, avapritinib overcomes intrinsic resistance to earlier-generation inhibitors, underscoring the importance of genomic profiling in therapy selection [181,182]. This paradigm highlights a shift from broad-spectrum multikinase inhibition, as seen with sunitinib and regorafenib, toward precision therapeutics tailored to specific molecular drivers, thereby enhancing efficacy and minimizing off-target toxicity [183,184].

Combination therapy represents another major opportunity. Preclinical and early-phase clinical studies suggest that pairing TKIs with immune checkpoint inhibitors, cytotoxic chemotherapies, or other targeted agents may achieve synergistic antitumor effects [185,186,187]. In mCRC, regorafenib combined with PD-1 inhibitors has shown promise in microsatellite-stable tumors that are otherwise resistant to immunotherapy [188,189]. Similarly, rational combinations of sunitinib or regorafenib with other antiangiogenic or MAPK-pathway inhibitors may overcome adaptive resistance and improve long-term disease control [190,191].

Biomarker-driven therapy and real-time pharmacodynamic monitoring are also emerging as critical tools to optimize treatment. Treatment-emergent adverse events, such as hypertension with sunitinib or hand-foot skin reaction with regorafenib, have been correlated with improved outcomes, suggesting potential use as mechanism-based biomarkers for patient stratification [192,193]. In avapritinib-treated GIST, rapid objective responses in PDGFRA D842V-mutant tumors serve as predictive biomarkers for durable disease control, reinforcing the role of early genomic and phenotypic assessment in guiding therapy [194].

Advances in drug formulation and dosing strategies provide additional opportunities to enhance tolerability and patient adherence. Intermittent dosing schedules, dose-escalation strategies, and pharmacokinetic-guided administration of TKIs are increasingly being explored to mitigate toxicity without compromising efficacy [195]. Such strategies are particularly relevant for chronic therapy settings, as these agents are often administered long-term for disease stabilization.

The integration of TKIs into earlier lines of therapy and adjuvant settings is another growing area of investigation. Clinical trials are evaluating sunitinib and regorafenib in neoadjuvant or adjuvant settings for high-risk GIST and advanced CRC, aiming to prevent recurrence and improve long-term survival [196,197]. Avapritinib may similarly expand into earlier treatment lines for patients with PDGFRA-mutant GIST, potentially reshaping the standard-of-care landscape [198].

Finally, advances in understanding resistance mechanisms are driving the development of next-generation inhibitors and rational sequencing strategies. Secondary mutations in KIT and PDGFRA, compensatory activation of parallel signaling pathways, and tumor microenvironment-mediated resistance continue to limit long-term efficacy of current TKIs. Research focused on overcoming these mechanisms through structural-guided drug design, combination therapy, and biomarker-informed therapy selection represents a fertile area for future innovation [199,200].

The evolving landscape of second- and third-generation TKIs in gastrointestinal malignancies is characterized by precision targeting, rational combination therapy, biomarker-guided strategies, and adaptive dosing. These trends highlight an ongoing shift toward highly individualized treatment paradigms, offering opportunities to improve outcomes, reduce toxicity, and expand the therapeutic potential of sunitinib, regorafenib, and avapritinib. Continued integration of molecular insights, translational research, and clinical innovation promises to further refine the role of TKIs in the management of gastrointestinal malignancies [201,202]. Table 8 presents contemporary management of gastrointestinal malignancies—gastrointestinal stromal tumours: current methods of treatment.

## 6. Conclusions

Second- and third-generation TKIs such as sunitinib, regorafenib, and avapritinib have substantially reshaped the therapeutic landscape of gastrointestinal malignancies, particularly in GIST and mCRC. These agents exemplify the evolution from broad-spectrum multikinase inhibition toward precision-targeted therapy, where molecular profiling and mutation-specific design guide optimal treatment selection.

Sunitinib remains a cornerstone for patients with imatinib-resistant GIST, with a well-characterized safety profile and documented efficacy in targeting VEGFR, PDGFR, and KIT-driven pathways. Regorafenib, as a multikinase inhibitor, extends therapeutic options for refractory mCRC and GIST, demonstrating the value of multitargeted inhibition of angiogenic, stromal, and oncogenic pathways. Avapritinib, a third-generation selective TKI, represents a paradigm shift by specifically addressing PDGFRA D842V mutations, a population historically refractory to first- and second-generation TKIs.

Emerging trends highlight several opportunities to further enhance patient outcomes. These include combination strategies with immune checkpoint inhibitors or pathway-specific agents, biomarker-guided therapy for individualized dose optimization and toxicity management, and rational sequencing to overcome acquired resistance. Precision dosing, real-time pharmacodynamic monitoring, and predictive adverse event biomarkers further underscore the importance of personalized therapy in maximizing efficacy while minimizing toxicity.

Despite these advances, challenges remain. Resistance mechanisms, including secondary mutations, compensatory signaling, and tumor microenvironment adaptation, continue to limit long-term efficacy. Ongoing translational research and clinical trials investigating novel combinations, early-line interventions, and mutation-driven strategies are critical to addressing these gaps and expanding the therapeutic potential of TKIs.

In conclusion, the integration of molecularly targeted TKIs into contemporary treatment paradigms exemplifies the shift toward personalized oncology in gastrointestinal malignancies. Continued innovation in drug design, patient stratification, and combination therapy is likely to refine their role, improve long-term outcomes, and broaden the applicability of TKIs in both GIST and other gastrointestinal cancers. The evolving landscape underscores a promising future where mutation-specific, mechanism-informed therapy becomes standard practice, optimizing efficacy while reducing adverse effects.

## Figures and Tables

**Table 1:** First-, second-, and third-generation TKIs used in gastrointestinal malignancies and related cancers, where AXL—AXL receptor tyrosine kinase; BCR-ABL—fusion oncoprotein driving CML; CML—chronic myeloid leukemia; CSF-1R—colony-stimulating factor 1 receptor; FGFR—fibroblast growth factor receptor; FLT3—FMS-like tyrosine kinase 3; GIST—gastrointestinal stromal tumor; HCC—hepatocellular carcinoma; KIT—KIT receptor tyrosine kinase; MET—MET (HGF) receptor tyrosine kinase; mCRC—metastatic colorectal cancer; PDGFRA—platelet-derived growth factor receptor alpha; PDGFR—platelet-derived growth factor receptor; PDGFRα/β—PDGF receptors alpha and beta; pNETs—pancreatic neuroendocrine tumors; RAF—RAF kinase family; RCC—renal cell carcinoma; RET—RET receptor tyrosine kinase; TIE2—angiogenesis receptor tyrosine kinase; TKIs—tyrosine kinase inhibitors; VEGFR1–3—vascular endothelial growth factor receptors 1–3.

Generation	Drug Name (Brand Name)	Key Targets	Key Clinical Trial(s)/Registry ID	No. of Patients	Key Efficacy Outcomes	Common Clinically Relevant Adverse Events	Clinical Use
First	Imatinib (Gleevec^®^)	KIT, PDGFR, BCR-ABL	B2222 (NCT00004005); S0033 (NCT00009906)	~147; ~746	Median PFS: ~18–24 mo; Median OS: >50 mo	Edema, nausea, diarrhea, fatigue, cytopenias	GIST, CML [18,19,20]
Second	Sunitinib (Sutent^®^)	KIT, VEGFR1-3, PDGFRα/β, FLT3, CSF-1R, RET	Phase III trial (NCT00075218)	312	Median PFS: 6.3 mo vs. 1.5 mo (placebo)	Hypertension, fatigue, hand–foot syndrome, cytopenias	Imatinib-resistant/intolerant GIST, RCC, pNETs [27]
Second	Regorafenib (Stivarga^®^)	VEGFR1-3, TIE2, PDGFRβ, FGFR, KIT, RET, RAF	GRID (NCT01271712)	199	Median PFS: 4.8 mo vs. 0.9 mo (placebo)	Hand–foot skin reaction, hypertension, diarrhea, fatigue	Refractory mCRC, GIST after imatinib/sunitinib, HCC [28]
Second	Sorafenib (Nexavar^®^)	RAF, VEGFR2-3, PDGFRβ, KIT, FLT3, RET	Phase II studies (NCT00474994)	~40–50	Median PFS: ~5–6 mo (off-label)	Rash, diarrhea, hypertension, fatigue	Advanced HCC, RCC, thyroid cancer [29]
Second	Pazopanib (Votrient^®^)	VEGFR1-3, PDGFRα/β, KIT, FGFR	PAZOGIST (NCT01323400)	81	Median PFS: 3.4 mo vs. 2.3 mo (BSC)	Hypertension, diarrhea, hepatotoxicity	Soft tissue sarcoma, RCC [30]
Second	Cabozantinib (Cabometyx^®^, Cometriq^®^)	MET, VEGFR2, RET, AXL, KIT	CaboGIST (NCT02216578)	50	Median PFS: 5.5 mo	Diarrhea, fatigue, hypertension, mucositis	RCC, HCC, medullary thyroid carcinoma [31]
Second	Axitinib (Inlyta^®^)	VEGFR1-3, PDGFR, KIT	Phase II trial (NCT01424436)	30	Median PFS: ~6 mo	Hypertension, fatigue, diarrhea	RCC [32]
Third	Avapritinib (Ayvakit^®^)	KIT, PDGFRA (highly selective for PDGFRA D842V)	NAVIGATOR (NCT02508532); VOYAGER (NCT03465722)	>250	ORR: ~88% (PDGFRA D842V); Median PFS: ~24 mo	Cognitive effects, edema, nausea, anemia	PDGFRA D842V-mutant GIST [47]
Third	Ripretinib (Qinlock^®^)	KIT, PDGFRA (activation-loop & gatekeeper mutations)	GRID (NCT03353753)	129	Median PFS: 6.3 mo vs. 1.0 mo (placebo)	Alopecia, fatigue, myalgia, hand–foot syndrome	Advanced GIST after ≥3 TKIs [55]

**Table 2:** TEAE and management strategies for sunitinib, where ACE-I—Angiotensin-Converting Enzyme Inhibitor; ARB—Angiotensin Receptor Blocker; BP—Blood Pressure; CBC—Complete Blood Count; G-CSF—Granulocyte Colony-Stimulating Factor; GI—Gastrointestinal; HF—Heart Failure; LFTs—Liver Function Tests; LVEF—Left Ventricular Ejection Fraction; TSH—Thyroid-Stimulating Hormone; VEGF—Vascular Endothelial Growth Factor.

Adverse Event	Description/Notes	Dose-Limiting Toxicity (DLT)	Manageable Chronic Effect	Management Strategies
Fatigue [70,85]	Very common; may worsen with prolonged therapy	Grade ≥3 limiting daily activities	Mild/moderate, persistent	Evaluate for anemia or thyroid dysfunction Encourage light exercise Treat hypothyroidism if present Dose interruption/reduction for grade ≥ 3
Hypertension [27]	Often appears early due to VEGF pathway inhibition	Uncontrolled grade ≥ 3	Mild/moderate elevations	Monitor BP closely, weekly during cycle 1 Initiate/adjust antihypertensive therapy Dose interruption/reduction for uncontrolled grade ≥ 3
Hand–Foot Syndrome [86]	Erythema, pain, peeling on palms/soles	Persistent grade ≥ 2 impacting ADLs	Mild redness/tingling	Urea-based emollients, avoid friction Topical corticosteroids and analgesics Interrupt or reduce dose for persistent grade ≥ 2
Mucositis/Stomatitis [86]	Oral soreness, ulceration common	Grade ≥ 3 limiting oral intake	Mild discomfort	Saline/baking soda rinses Avoid irritant foods Topical anesthetics/corticosteroids Dose modification for grade ≥ 3
Diarrhea [27]	Frequent gastrointestinal toxicity	Grade ≥ 3 dehydration or electrolyte imbalance	Mild/moderate loose stools	Loperamide and hydration Dietary modification Dose interruption for grade ≥ 3
Nausea/Vomiting [87]	Usually mild to moderate	Persistent grade ≥ 3 affecting intake	Mild/moderate	Antiemetics Small, frequent meals
Anorexia/Weight Loss [87]	Often due to taste changes and GI toxicity	Severe weight loss or malnutrition	Mild decrease in appetite	Nutritional support Manage reversible contributors
Hypothyroidism [85]	Very common with chronic sunitinib exposure	Severe symptomatic hypothyroidism	Mild/subclinical	Monitor TSH every 4–6 weeks Start levothyroxine when indicated
Hematologic Toxicity [33]	Neutropenia, thrombocytopenia, anemia are dose-related	Grade ≥ 3 cytopenias	Mild/moderate decreases	Regular CBC monitoring G-CSF for severe neutropenia Transfusions if needed Dose adjustment for grade ≥ 3 cytopenias
Cardiotoxicity (LVEF decline, HF) [82]	Less common but clinically important	Symptomatic heart failure	Asymptomatic LVEF decline	Baseline and periodic echocardiography Treat with ACE-I and/or β-blockers Discontinue for symptomatic HF
Proteinuria/Nephrotic Syndrome [88]	VEGF-related renal toxicity	Nephrotic syndrome	Mild proteinuria	Regular urinalysis Interrupt/stop therapy for nephrotic syndrome
Hepatotoxicity [89]	Elevated AST/ALT; rare severe liver injury	Grade ≥ 3 AST/ALT elevation	Mild/moderate LFT elevation	Frequent LFTs Hold or reduce dose for grade ≥ 3
Skin/Hair Pigment Changes [87]	Drug-related; usually benign	Rarely dose-limiting	Cosmetic only	Reassurance; no intervention
Hypoglycemia [90]	Observed particularly in diabetics	Severe, symptomatic hypoglycemia	Mild glucose fluctuations	Monitor glucose closely Adjust antidiabetic medications

**Table 3:** Major pivotal clinical trials of sunitinib, where EAP—Expanded Access Program; FDA—U.S. Food and Drug Administration; GIST—Gastrointestinal Stromal Tumor; IFN-α—Interferon-Alpha; mRCC—Metastatic Renal Cell Carcinoma; NET—Neuroendocrine Tumor; ORR—Objective Response Rate; OS—Overall Survival; PFS—Progression-Free Survival; PK/PD—Pharmacokinetics/Pharmacodynamics; pNET—Pancreatic Neuroendocrine Tumor; TTP—Time to Progression.

Trial/Study	Indication	Design	Key Outcomes
Phase III Sunitinib vs. IFN-α [27]/NCT00083889	Metastatic renal cell carcinoma (mRCC)	Randomized, open-label Phase III	Improved PFS (11 vs. 5 months), higher objective response rate
Phase III Second-line GIST After Imatinib Failure [33]/NCT00075218	Gastrointestinal stromal tumor (GIST)	Randomized, double-blind Phase III	Significant improvement in TTP and OS; established second-line standard
Expanded Access Program (EAP) in mRCC [87]/NCT00333866	Metastatic RCC	Global, non-interventional expanded-access cohort	Confirmed efficacy and safety in real-world population
Phase II Pancreatic Neuroendocrine Tumor Trial [74]/NCT00428597	Pancreatic NET (pNET)	Randomized, double-blind, placebo-controlled	Improved PFS (11.4 vs. 5.5 months), ORR 9.3% → FDA approval
Dose-Schedule Optimization Study [87]/NCT00112661	RCC, GIST (safety and PK/PD)	Multicenter safety and dosing study	Identified 4/2 schedule tolerability; described safety profile
Long-term Outcomes in mRCC [97]/(pooled analysis, not a standalone trial)	Metastatic RCC	Pooled analysis of Phase II/III trials	Median OS 26–29 months; durable responses

**Table 4:** TEAE and management strategies for regorafenib, where AE—Adverse Event; BP—Blood Pressure; HFSR—Hand–Foot Skin Reaction; LFT—Liver Function Test; TEAE—Treatment-Emergent Adverse Event.

Adverse Event	Description	Dose-Limiting Toxicity (DLT)	Manageable Chronic Effect	Recommended Management Strategy
Hand–Foot Skin Reaction (HFSR) [28,130]	Palmar–plantar erythema, pain, hyperkeratosis	Persistent grade ≥ 2 limiting daily activities	Mild erythema, tingling, hyperkeratosis	Prophylactic urea-based creams; reduce friction; dose interruption or reduction if grade ≥ 2
Hypertension [131,132]	Early-onset elevation in blood pressure	Uncontrolled grade ≥ 3	Mild/moderate elevations	Weekly BP monitoring during first cycle; initiate antihypertensives; dose modification if uncontrolled
Fatigue [28,133]	Common nonspecific symptom	Grade ≥ 3 limiting daily activities	Mild/moderate, persistent fatigue	Rule out reversible causes; adjust dosing; encourage energy-conservation measures
Diarrhea [28,131]	Increased stool frequency; risk of dehydration	Grade ≥ 3 dehydration or electrolyte imbalance	Mild/moderate loose stools	Loperamide; oral hydration; dietary adjustments; dose reduction for grade ≥ 3
Hepatotoxicity [132,134,135]	Elevated liver enzymes or bilirubin	Grade ≥ 3 AST/ALT elevation or bilirubin	Mild/moderate LFT elevations	Baseline and frequent LFT monitoring; hold drug for grade ≥ 3; resume at reduced dose when resolved
Mucositis/Stomatitis [130,133]	Oral pain, ulcers	Grade ≥ 3 limiting oral intake	Mild oral soreness	Saline rinses; topical analgesics; avoid irritants; dose reduction if persistent
Rash/Desquamation [131,133]	Erythematous or exfoliative eruptions	Severe or symptomatic grade ≥ 3	Mild erythema, scaling	Emollients; topical corticosteroids; consider dose adjustments
Anorexia/Weight Loss [28,134,135]	Reduced appetite and nutritional decline	Severe weight loss or malnutrition	Mild decrease in appetite	Nutritional counseling; treat nausea; consider dose modification

**Table 5:** Major pivotal clinical trials of regorafenib, where GIST—Gastrointestinal Stromal Tumor; HCC—Hepatocellular Carcinoma; mCRC—Metastatic Colorectal Cancer; OS—Overall Survival; PFS—Progression-Free Survival.

Trial	Indication	Study Design	Key Outcomes
CORRECT [28]/NCT01103323	Metastatic colorectal cancer (mCRC)	Phase III, randomized, placebo-controlled	Improved OS and PFS vs. placebo in previously treated mCRC
CONCUR [142]/NCT01584830	Asian population with mCRC	Phase III, randomized, placebo-controlled	Significant OS benefit; stronger effect size than CORRECT due to less prior therapy
GRID [38]/NCT01271712	Gastrointestinal stromal tumor (GIST)	Phase III, randomized, placebo-controlled	Significant PFS improvement in patients progressing on imatinib and sunitinib
RESORCE [110]/NCT01774344	Hepatocellular carcinoma (HCC)	Phase III, randomized, placebo-controlled	Improved OS in patients previously treated with sorafenib
ReDOS [143]/NCT02368886	mCRC (dose-optimization study)	Phase II, randomized, strategy trial	Weekly dose-escalation strategy improved tolerability and allowed more patients to start cycle 3
REGISTRI [57]/NCT02638766	GIST (real-world, post-imatinib/sunitinib)	Observational, multicenter	Confirms efficacy and tolerability across diverse populations
Ongoing Combination Study [58,144]/NCT04200404, NCT03475953	GIST, regorafenib ± other agents (CS1001, avelumab)	Phase I/II	Investigating combination/synergistic late-line strategies
Other Relevant Non-Combination Ongoing Studies [108,145]/NCT06087263, NCT02889328	The efficacy of regorafenib as a single agent in specific GIST mutation subsets (KIT Exon 17, 18, or 14 mutation and SDHB-deficient) in the post-imatinib second-line setting. Continuous dosing schedule of regorafenib (100 mg daily) in patients with TKI-refractory GISTs, aiming to prevent disease flare during off-treatment periods of the standard intermittent schedule	Phase II	Investigating late-line strategies

**Table 6:** TEAE and management strategies for avapartinib, where AE—Adverse Event; CBC—Complete Blood Count; GI—Gastrointestinal; GIST—Gastrointestinal Stromal Tumor; TEAE—Treatment-Emergent Adverse Event.

Adverse Event	Description	Dose-Limiting Toxicity (DLT)	Manageable Chronic Effect	Recommended Management Strategy
Cognitive Effects [16,169]	Memory impairment, confusion, mood changes	Severe impairment affecting daily functioning	Mild memory or attention changes	Baseline cognitive assessment; dose interruption or reduction; consider neurocognitive evaluation if persistent
Edema [16]	Periorbital or peripheral swelling	Severe, symptomatic swelling requiring hospitalization or limiting function	Mild peripheral or periorbital edema	Salt restriction; compression garments; diuretics if symptomatic; dose adjustment if severe
Fatigue [170]	Generalized tiredness	Grade ≥ 3 limiting daily activities	Mild/moderate, persistent fatigue	Evaluate reversible causes; energy-conservation measures; dose modification for grade ≥ 3
Gastrointestinal Toxicity (Nausea, Vomiting) [169,170]	Common early-onset GI symptoms	Persistent grade ≥ 3 affecting intake or hydration	Mild/moderate nausea or vomiting	Prophylactic or PRN antiemetics; dietary adjustments; consider dose reduction if persistent
Hematologic Toxicity [16,171]	Anemia, thrombocytopenia	Grade ≥ 3 cytopenias	Mild/moderate decreases in blood counts	Routine CBC monitoring; transfusions if indicated; dose interruption for grade ≥ 3
Intracranial Bleeding [16]	Rare but serious AE	Symptomatic intracranial hemorrhage	N/A (rare; generally DLT only)	Avoid anticoagulation when possible; monitor neurologic symptoms; immediate drug interruption if suspected
Photosensitivity/Rash [169]	Skin erythema or reactions to sunlight	Severe, symptomatic rash or desquamation	Mild erythema	Sun protection; topical corticosteroids; dose modification for grade ≥ 3
Weight Loss/Anorexia [170]	Reduced appetite and weight decline	Severe weight loss or malnutrition	Mild decrease in appetite	Nutritional counseling; treat contributing symptoms; dose modification if significant

**Table 7:** Major pivotal clinical trials of avapartinib, where advSM—Advanced Systemic Mastocytosis); GIST—Gastrointestinal Stromal Tumor; ORR—Overall Response Rate; PDGFRA—Platelet-Derived Growth Factor Receptor Alpha; PFS—Progression-Free Survival; TKI—Tyrosine Kinase Inhibitor.

Trial	Indication	Study Design	Key Outcomes
NAVIGATOR [47]/NCT02508532	Unresectable or metastatic GIST with PDGFRA exon 18 mutations (incl. D842V)	Phase I, open-label, dose-escalation/expansion	High ORR (notably >80% in PDGFRA D842V); durable responses; manageable safety profile
VOYAGER [172]/NCT03465722	Advanced GIST after ≥2 prior TKIs (imatinib, sunitinib ± regorafenib)	Phase III, randomized, avapritinib vs. regorafenib	Did not meet primary PFS endpoint overall; certain molecular subgroups showed activity
CS3007-101, NCT04254939–phase 1/2 (China bridging) trials/Early-phase supporting study [160]	Advanced solid tumors, including GIST (unresectable or metastatic GISTs)	Phase I, dose-finding	Established recommended dose; characterized safety and early antitumor activity
PATHFINDER [177]/NCT03580655	Advanced systemic mastocytosis (advSM)	Phase II, open-label	High response rates across advSM subtypes; marked reductions in mast-cell burden and symptoms

**Table 8:** Contemporary management of gastrointestinal malignancies—gastrointestinal stromal tumours: current methods of treatment, where: CT—Computed Tomography; DVT—Deep Venous Thrombosis; GIST—Gastrointestinal Stromal Tumour; MRI—Magnetic Resonance Imaging; PDGFRA—Platelet-Derived Growth Factor Receptor Alpha; PFS—Progression-Free Survival; R0—Complete resection with negative margins; RFS—Recurrence-Free Survival; SDH—Succinate Dehydrogenase; TKI—Tyrosine Kinase Inhibitor; VEGF—Vascular Endothelial Growth Factor.

Treatment/Strategy	Setting/Indication	Key Evidence/Trial (Summary)	Notes/Practical Considerations
Surgery (complete resection, R0)	Localized, resectable GIST	Standard of care for primary localized GIST—complete resection with negative margins is goal; lymphadenectomy usually not required [203,204].	Consider tumor rupture risk; refer to sarcoma/GIST center; perioperative planning with mutation results if neoadjuvant considered.
Adjuvant imatinib 400 mg daily	High-risk resected GIST (per risk criteria)	SSGXVIII/AIO randomized trial: 36 months > 12 months adjuvant imatinib—improved recurrence-free survival and overall survival in high-risk patients [23,204].	Recommended for high-risk patients with imatinib-sensitive mutations (KIT exon 11); duration typically 3 years for high-risk patients; assess tolerability and mutational status.
Neoadjuvant (preoperative) imatinib	Large primary tumor or borderline resectable disease to enable organ-sparing surgery	Multiple series and prospective studies show tumor downsizing and increased resectability; individualized duration (commonly 6–12 mo) guided by response and operability [204,205].	Use when resection would be morbid; require early mutation testing to predict sensitivity; monitor response with CT/MRI.
Imatinib for advanced/unresectable/metastatic GIST	First-line systemic therapy for advanced disease	Pivotal trials and long-term experience show high response and durable disease control with imatinib (400 mg daily; dose escalation to 800 mg for some KIT exon 9 mutations) [19,204].	Obtain KIT/PDGFRA mutation testing before or at start; consider dose escalation for KIT exon 9; monitor toxicity and response.
Sunitinib (multitargeted TKI)	Second-line therapy after imatinib intolerance/progression	Randomized trials established sunitinib as standard second-line therapy with PFS benefit after imatinib failure [33].	Active across certain secondary KIT mutations; dose schedule options (e.g., 50 mg 4/2 or continuous lower dose)—tailor to tolerability.
Regorafenib	Third-line therapy after imatinib + sunitinib failure	GRID phase 3 trial: regorafenib improved PFS vs. placebo in patients progressing on imatinib and sunitinib [38].	Consider as standard third-line therapy systemic agent; monitor for hand–foot syndrome, hypertension, hepatic toxicity.
Ripretinib	Fourth-line (or later) therapy after ≥3 prior TKIs (including imatinib)	INVICTUS phase 3: ripretinib improved PFS and OS vs. placebo in heavily pretreated patients [55]	Broad-spectrum switch-control TKI with activity against many resistant mutations; approved for later-line therapy.
Avapritinib	Advanced/metastatic GIST with PDGFRA exon 18 (including D842V) mutation	NAVIGATOR trial (phase 1/1b and follow-up) demonstrated high response rates in PDGFRA D842V-mutant GIST—led to regulatory approvals for this molecular subgroup [47]	Mandatory mutation testing to identify PDGFRA D842V; avapritinib is preferred for this genotype. Monitor for cognitive and intracranial hemorrhage warnings.
Mutation testing (KIT, PDGFRA, SDH, NF1, BRAF, etc.)	All newly diagnosed GIST (ideally before systemic therapy)	Guidelines (ESMO, NCCN, GEIS/SEOM) recommend routine molecular testing because mutation status guides choice/dose of TKI and prognosis [203,204].	Send tumor (or ctDNA when tissue not available) for exon-level KIT/PDGFRA testing; inform adjuvant/neoadjuvant and systemic therapy decisions.
Local therapy for oligoprogression/limited disease	Selected patients on TKI with limited progressing lesions	Surgical metastasectomy or local ablative procedures (radiofrequency ablation, stereotactic radiotherapy) can be considered to control progressing clones while maintaining systemic TKI [203,204].	Multidisciplinary decision; continue systemic TKI unless contraindicated; reserve for selected pts in specialized centers.
Surveillance/follow-up imaging	Post-resection or while on systemic therapy	Guideline-based intervals depend on risk group and therapy—higher-risk patients require more frequent CT/MRI [203,204].	Use contrast-enhanced CT or MRI; tailor to risk and clinical course; long-term follow-up often required.

## Data Availability

Data sharing is not applicable to this article.

## Abbreviations

The following abbreviations are used in this manuscript:

ACE-I	Angiotensin-Converting Enzyme Inhibitor
advSM	Advanced Systemic Mastocytosis
AE	Adverse Event
AKT	Protein Kinase B
ARB	Angiotensin Receptor Blocker
ALK	Anaplastic Lymphoma Kinase
AXL	AXL Receptor Tyrosine Kinase
BCR-ABL	Fusion Oncoprotein Driving Chronic Myeloid Leukemia (CML)
BP	Blood Pressure
B-RAF	Serine/Threonine-Protein Kinase B-RAF
CBC	Complete Blood Count
C-KIT	Mast/Stem Cell Growth Factor Receptor
CML	Chronic Myeloid Leukemia
CSF-1R	Colony-Stimulating Factor 1 Receptor
CT	Computed Tomography
DVT	Deep Venous Thrombosis
EAP	Expanded Access Program
EGFR	Epidermal Growth Factor Receptor
ERK	Extracellular Signal-Regulated Kinase
FDA	U.S. Food and Drug Administration
FGFR	Fibroblast Growth Factor Receptor
FLT3	FMS-Like Tyrosine Kinase 3
G-CSF	Granulocyte Colony-Stimulating Factor
GI	Gastrointestinal
GIST	Gastrointestinal Stromal Tumor
HCC	Hepatocellular Carcinoma
HER	Human Epidermal Growth Factor Receptor
HF	Heart Failure
HFSR	Hand–Foot Skin Reaction
IFN-α	Interferon-Alpha
KIT	KIT Receptor Tyrosine Kinase
LFT/LFTs	Liver Function Test / Liver Function Tests
LVEF	Left Ventricular Ejection Fraction
MEK	Mitogen-Activated Protein Kinase
MET	MET (HGF) Receptor Tyrosine Kinase
mCRC	Metastatic Colorectal Cancer
MRI	Magnetic Resonance Imaging
mRCC	Metastatic Renal Cell Carcinoma
NET	Neuroendocrine Tumor
ORR	Objective Response Rate / Overall Response Rate
OS	Overall Survival
PDGFRα(A)/β(B)	Platelet-Derived Growth Factor Receptors Alpha and Beta
PFS	Progression-Free Survival
PI3K	Phosphoinositide 3-Kinase
PK/PD	Pharmacokinetics/Pharmacodynamics
pNET/pNETs	Pancreatic Neuroendocrine Tumor(s)
RAS	Rat Sarcoma Protein
RAF	Rapidly Accelerated Fibrosarcoma Kinase
RCC	Renal Cell Carcinoma
RET	RET Receptor Tyrosine Kinase
R0	Complete Resection with Negative Margins
RFS	Recurrence-Free Survival
ROS1	Proto-Oncogene Tyrosine-Protein Kinase ROS
SDH	Succinate Dehydrogenase
TEAE	Treatment-Emergent Adverse Event
TIE2	Angiogenesis Receptor Tyrosine Kinase
TKI/TKIs	Tyrosine Kinase Inhibitor(s)
TIE2	Tunica Interna Endothelial Cell Kinase 2
TRKs	Tropomyosin Receptor Tyrosine Kinases
TSH	Thyroid-Stimulating Hormone
TTP	Time to Progression
VEGF	Vascular Endothelial Growth Factor
VEGFR1–3	Vascular Endothelial Growth Factor Receptors 1–3
